# METHODS, THEORY, AND INNOVATION A BAYESIAN ANALYSIS OF EVIDENCE IN SUPPORT OF THE NULL HYPOTHESIS IN GERONTOLOGICAL PSYCHOLOGY (OR LACK THEREOF)

**DOI:** 10.1093/geroni/igz038.2841

**Published:** 2019-11-08

**Authors:** Christopher Brydges, Allison A Bielak

**Affiliations:** Colorado State University, Fort Collins, Colorado, United States

## Abstract

Objective: Non-significant p values derived from null hypothesis significance testing do not distinguish between true null effects or cases where the data are insensitive in distinguishing the hypotheses. This study aimed to investigate the prevalence of Bayesian analyses in gerontological psychology, a statistical technique that can distinguish between conclusive and inconclusive non-significant results, by using Bayes factors (BFs) to reanalyze non-significant results from published gerontological research. Method: Non-significant results mentioned in abstracts of articles published in 2017 volumes of ten top gerontological psychology journals were extracted (N = 409) and categorized based on whether Bayesian analyses were conducted. BFs were calculated from non-significant t-tests within this sample to determine how frequently the null hypothesis was strongly supported. Results: Non-significant results were directly tested with Bayes factors in 1.22% of studies. Bayesian reanalyses of 195 non-significant t-tests found that only 7.69% of the findings provided strong evidence in support of the null hypothesis. Conclusions: Bayesian analyses are rarely used in gerontological research, and a large proportion of null findings were deemed inconclusive when reanalyzed with BFs. Researchers are encouraged to use BFs to test the validity of non-significant results, and ensure that sufficient sample sizes are used so that the meaningfulness of null findings can be evaluated.

